# Mortality assessment of patients with hepatocellular carcinoma according to underlying disease and treatment modalities

**DOI:** 10.1097/MD.0000000000005904

**Published:** 2017-03-03

**Authors:** Pegah Golabi, Sofie Fazel, Munkhzul Otgonsuren, Mehmet Sayiner, Cameron T. Locklear, Zobair M. Younossi

**Affiliations:** a Betty and Guy Beatty Center for Integrated Research, Inova Health System, Falls Church; b Center For Liver Disease, Department of Medicine, Inova Fairfax Hospital Falls Church, VA.

**Keywords:** hepatocellular carcinoma, liver transplantation, mortality, surgical resection

## Abstract

Hepatocellular carcinoma (HCC) is among the most common types of cancer. Liver transplantation (LT) and surgical resection (SR) are primary surgical treatment options for HCC.

The aim of the study was to assess mortality within 2 years postdiagnosis among patients with HCC according to their treatment modalities.

We examined data from the Surveillance, Epidemiology and End Results (SEER)-Medicare database between 2001 and 2009. SEER registries collect demographics, cancer stage and historical types, and treatments. Medicare claims include diagnoses, procedures, and survival status for each beneficiary. Patients with HCC were identified using the International Classification of Disease Oncology, Third Edition Site code C22.0 and Histology Code 8170-8175. Treatment modalities were LT, SR, or nonsurgical treatment.

Total of 11,187 cases was included (age at diagnosis: 72 years, 69% male, 67% White). HCC patients who underwent LT were younger (61 vs 71 years), sicker (presence of decompensated cirrhosis: 80% vs 23%), and less likely to die within 2 years (29% vs 44%, all *P* < 0.01), compared to SR patients. In multivariate analysis, older age (HR: 1.01 [95% CI = 1.01–1.01]), stage of HCC other than local (HR: 1.81[95%CI = 1.70–1.91]), and being treated with SR (HR: 1.95 [95%CI = 1.55–2.46]) were independent predictors of mortality within 2 years. Furthermore, the presence of decompensated cirrhosis (HR: 1.84 [95%CI = 1.73–1.96]) and alcoholic liver disease (HR: 1.19[95%CI = 1.11–1.28]) increased within 2 years mortality.

Mortality within 2 years postdiagnosis of HCC was significantly higher in patients treated with SR than LT.

## Abstract 2

Supplemental Digital Content is available in the text

## 1 Introduction

Cancer is among the leading causes of morbidity and mortality worldwide, accounting for 14 million new cases and 8.2 million deaths in 2012.^[1,2]^ Globally, liver cancer is the fifth most common type of cancer and third most common cause of cancer mortality.^[3]^ With an estimated 746,000 deaths in 2012, liver cancer is the second most common cancer-related deaths, worldwide.^[4]^ In the United States (US), according to the Surveillance, Epidemiology, and End Results Program (SEER) estimates, in 2015, liver cancer accounted for 2.2% of all new cancer cases and 4.2% of all cancer deaths.^[5]^ Although metastatic tumors are the most frequently seen type of cancer of the liver, hepatocellular carcinoma (HCC) is the most common primary liver cancer, accounting for nearly 80% of all primary liver cancers.^[6,7]^

In the United States, hepatocellular carcinoma has been recognized as the ninth leading cause of cancer-related deaths.^[7–10]^ Furthermore, HCC incidence and mortality rates have been increasing for decades.^[11,12]^ Unfortunately, HCC is typically diagnosed late in its course, with a median survival following diagnosis of approximately 6 to 20 months. In the United States, 2 years survival is less than 50% and 5-year survival is only 10%.^[13–15]^

The effective management of HCC involves a multidisciplinary approach, involving hepatologists, surgeons, radiologists, and liver transplant team. In this context, treatment modalities for HCC patients include surgical resection, radiofrequency ablation, microwave ablation, percutaneous ethanol or acetic acid injection, transarterial chemoembolization (TACE), liver transplantation (LT) and, rarely, systemic chemotherapy.^[16,17]^ The ideal treatment option for a specific patient with HCC is determined based on the burden of tumor and extent of underlying liver disease.

Typically, patients with early-stage HCC and cirrhosis are candidates for liver transplantation, whereas surgical resection remains the treatment of choice in patients without significant underlying liver disease.^[18,19]^ Nevertheless, there is still some ongoing debate about the best treatment option for patients with early stage HCC and well-compensated cirrhosis.^[20]^ Liver transplantation provides the benefit of removing the underlying diseased liver, but it is limited because of the donor organ availability. Although surgical resection maybe an option, it is limited by the high morbidity and mortality caused by hepatectomy in patients with underlying liver disease.^[21,22]^ Previous studies have clearly shown that the recurrence rates of HCC and overall survival varied according to the treatment option.^[20,21]^ In this study, our aim was to compare the short-term mortality among patients with HCC according to treatment modality.

## 2 Methods

### 2.1 Study design and population

A retrospective, cross-sectional study of the SEER-Medicare database files (Patient Entitlement and Diagnosis Summary File [PEDSF] for identifying HCC cases via linkage Medicare DENOM file for those who identified as a SEER case, further, DENOM linked Medicare Provider Analysis and Review file [MEDPAR], outpatient file, and Physician/Supplier bills file [NCH]) were used for this analysis between 2001 and 2009. Briefly, the US population-based SEER registries collect demographic, stage and historical type, and types of initial cancer treatments in PEDSF. Medicare files include claims (dates of service and codes for specific diagnoses and procedures using the International Classification of Disease, ninth revision [ICD-9-CM] codes, enrollment, and vital status) for each beneficiary that fee-for-service covered hospitalizations, outpatient, and physician services. In this study, SEER-Medicare data were used and the study was approved by the Institutional Review Board of Inova Fairfax Hospital; the approval number is: 13-.1432.

There were 20,409 HCC cases in SEER-Medicare, 2001–2009. The following cases were excluded: (1) missing information on date of HCC diagnosis, n = 233; (2) diagnosis confirmed only after autopsy or death, n = 312; (3) missing information on the Charlson Comorbidity Index (CCI), n = 5,714; (4) HIV infection, n = 210; (4) not continuously enrolled for at least 12-months on Medicare fee-for-services, n = 3,105; and (5) unknown information on the surgical resection, n = 193. The above excluded cases with missing information on CCI and not continuously enrolled on fee-for-service for at least 12-months had an average 65 years old, 76% male, 54% local staging HCC.

### 2.2 Outcome

The within 2 years survival rates were calculated for all patients diagnosed with HCC between 2001 and 2009 (followed up until February 8, 2012, with average follow-up 4 months [IQR: 1–12]).

### 2.3 Definitions of liver diseases

The following 4 categories of chronic liver diseases were identified using the ICD-9 codes: (1) *hepatic C virus (HCV)* with codes 070.7, 070.41, 070.44, 070.51, 070.54, V02.62; (2) *hepatitis B virus (HBV)* with codes 070.2, 070.3, 070.42, 070.52, V02.61; (3) *alcoholic liver disease* with codes 303, 291, 571,0, 571.1, 571.2, 571.3, 305.0, V11.3, V79.1; and (4*) nonviral and nonalcoholic cryptogenic liver disease* with codes 571.8, 571.9, 571.5. Moreover, we identified *decompensated hepatic cirrhosis* by codes 789.5 as ascites, 567.23 as spontaneous bacterial peritonitis, 456.0 as esophageal varices with bleeding, 456.2 as esophageal varices in disease classified elsewhere, code underlying cause are cirrhosis of liver and portal hypertension, and 572.2 as hepatic encephalopathy.

### 2.4 Definition of treatments

*Liver transplant (LT) recipients* were identified by ICD-9 codes V427, 505.1, 505.9 using MEDPAR, NCH, and Outpatient files. *Surgical resection (SR)* was defined by “sxprif1-sxprif10” (code “00” as No surgery and coded as 0; and codes 10–19 tumor destruction, 20–80 resection, or 90 surgery to the primary site as Yes performed SR and coded as 1) using PEDSF file. A usage of *transarterial chemoembolization (TACE)* (ICD-9 procedure codes 38.80, 38.86, 99.25 and CPT codes 37204, 75894, J9000, J9280, J9060, 96405, 96408, 96420, 96422, 96423, 96425, 96440, 96445, 96545, 96549, 0331, 0335 using Medicare MEDPAR, NCH, and Outpatient files.

### 2.5 Data analysis

**Figure 1:**
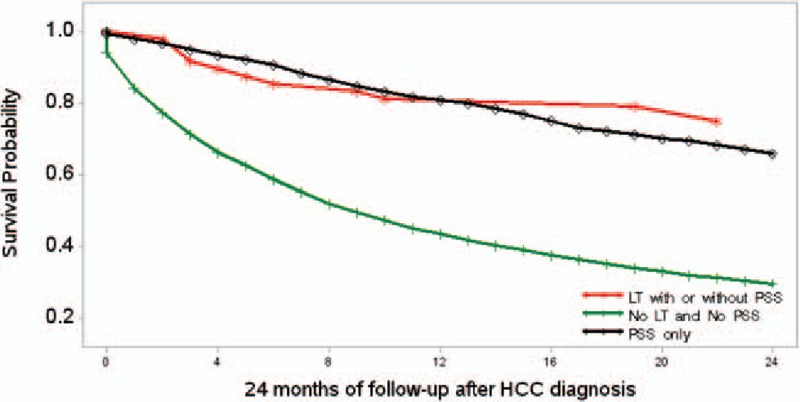
Kaplan–Meier survival curves for HCC patients by liver transplant and primary site surgery status in the subcohort. HCC = hepatocellular carcinoma.

All analyses were performed using SAS Version 9.3 (SAS Institute, Cary, NC). Baseline characteristics of study patients were presented by mean (standard deviation) for continuous variables and frequency (percentage) for categorical variables. Differences in categorical variables were examined using the CHISQ test and differences in continuous variables were examined using *ANOVA* by LT/SR status. Cox proportional hazard models were fitted to estimated univariate and multivariate adjusted hazards ratios (HRs) and 95% confidence intervals (CIs) for the associations of within 2 years mortality after diagnosis of HCC and LT/SR status and baseline characteristics. In order to compare within 2 years mortality between liver transplantation and surgical resection in patients with local HCC in the absence of decompensated cirrhosis and in the absence of primary tumor stage regional/distant/unstaged, a subcohort analysis was performed. In this sub cohort (n = 3523), due to the small sample size (n = 48) of LT, we examined the association between LT/SR status and within 2 years mortality only by Kaplan–Meier survival curves estimates (Fig. 1). We did not examine the adjusted association between within 2 years mortality and baseline characteristics while adjusting LT/SR status. All reported *P* values are 2-sided and defined as significant at the 5% level.

## 3 Results

### 3.1 General characteristics of study population

After inclusion and exclusion criteria, a total of 11,187 cases of HCC were enrolled in the study (Table 1). Among the study group, 302 patients with HCC received liver transplantation (LT), 2243 patients with HCC received only surgical resection (SR) and 8642 patients with HCC received neither LT nor SR. For the entire group, mean age at HCC diagnosis was 72 ± 10 years, 69% men, and 67% White. Furthermore, 52% of patients had HCV, 9% had HBV, 21% had alcoholic liver disease, and 19% had nonviral and nonalcoholic/cryptogenic liver disease. From the entire group, 34% of patients of HCC had decompensated cirrhosis and 69% had a mean CCI of 2+ and 27% have been treated with TACE. Also, 53% of HCC patients had local disease, whereas 47% had distant disease/unstaged tumor site.

**Table 1:**
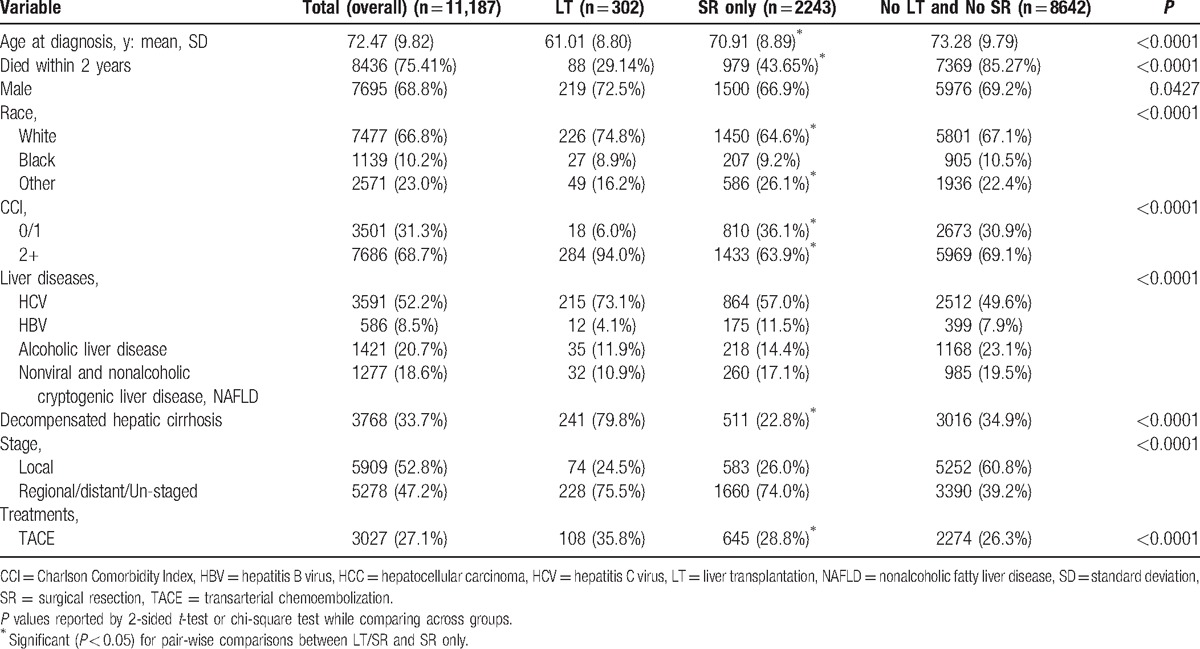
Characteristics of study by liver transplantation (LT) and surgical resection (SR) status in HCC, SEER-Medicare, 2001–2009.

### 3.2 Comparison of liver transplant recipients to the patients who were treated with surgical resection

**Figure 2:**
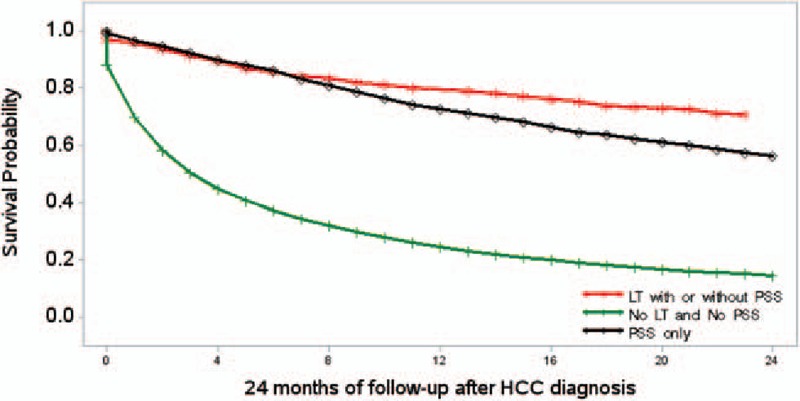
Kaplan–Meier survival curves for HCC patients by liver transplant and primary site surgery status in the main cohort. HCC = hepatocellular carcinoma.

Mean age at HCC diagnosis was significantly higher in SR only group than the LT group (71 vs 61 years, *P* < 0.0001) (Table 1). Mortality within 2 years of diagnosis was significantly higher in the SR group than patients receiving LT (29% vs 44%, *P* < 0.0001). Kaplan–Meier survival curve for mortality for HCC patients who were treated with different modalities are shown in Fig. 2. Even after pair-wise matched analysis 2 years mortality was significantly higher in the SR group than the LT group (See Table, Supplemental content which illustrates pair-wise matched analysis of SR and LT groups). Also, 73% of HCC patients who received LT had HCV infection and 12% had alcoholic liver disease. In contrast, 57% of HCC patients treated with surgical resection had HCV, whereas 17% had nonviral and nonalcoholic/cryptogenic liver disease. As expected, the rate of decompensated cirrhosis was higher in the LT group than those treated with SR (80% vs 23%, *P* < 0.05). The prevalence of tumor site stage were similar in the both groups, as 76% of LT group and 74% of SR only group had local disease (*P* > 0.05). Finally, the proportion of patients receiving TACE was significantly higher in the LT group than SR only group (36% vs 29%, *P* < 0.0001).

### 3.3 Contributors to mortality within 2 years after diagnosis of hepatocellular carcinoma

Univariate analysis revealed that the age increased mortality within the first 2 years after diagnosis (HR: 1.02[95% CI = 1.02–1.02]) (Table 2). Looking at the underlying liver disease, compared to patients with HCV infection, patients with HBV infection were less likely to die within 2 years (HR: 0.82[95% CI = 0.73–0.92]), whereas the risk of mortality within 2 years of diagnosis was higher in patients with alcoholic liver disease (HR: 1.59 [95%CI = 1.49–1.71]) and nonviral/nonalcoholic cryptogenic liver disease (HR: 1.30[95%CI = 1.21–1.40]). Also, the presence of decompensated cirrhosis significantly increased mortality within the 2 years (HR: 1.82[95%CI = 1.72–1.92]). Finally, as compared to the LT group, SR only group (HR: 1.56[95%CI = 1.26–1.94]) and patients who did not receive LT or SR (HR: 5.88[95%CI = 4.76–7.26]) had significantly higher mortality within 2 years of diagnosis.

**Table 2:**
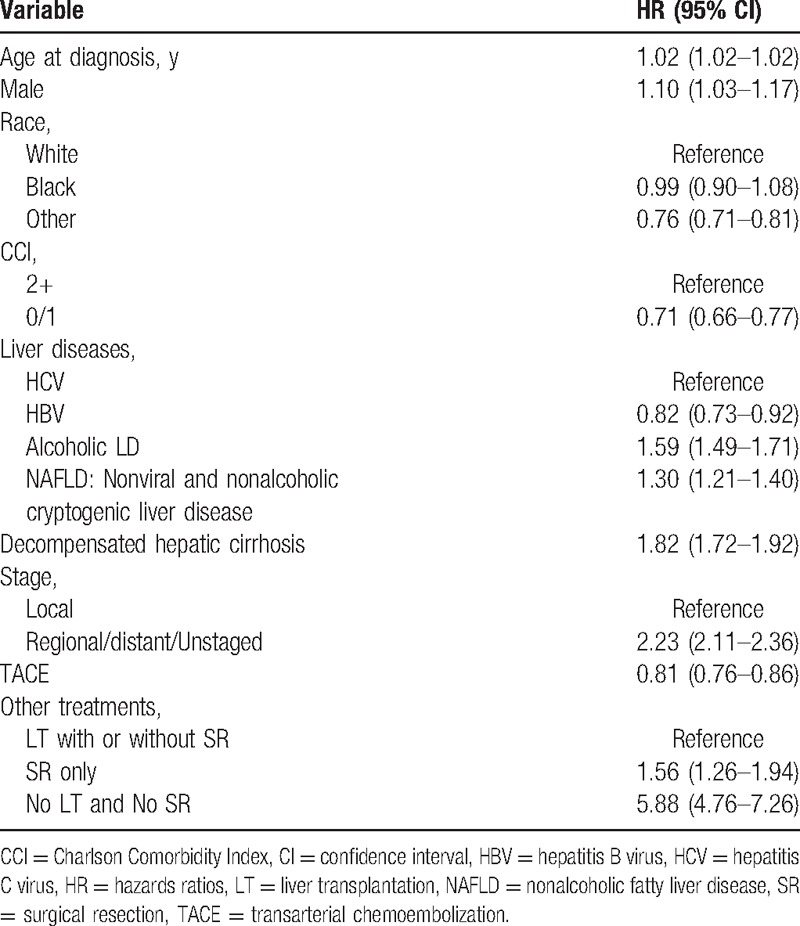
Univariate adjusted hazards ratios (HRs) with 95% confidence intervals (CIs) for within 2 years mortality.

Most of those predictors of mortality within the 2 years after diagnosis remained significant and did not change the directions and magnitudes of associations in multivariate analysis (Table 3). As compared to patients who underwent LT, individuals who received SR only (HR: 1.95[95%CI = 1.55–2.46]) and patients who did not receive LT or SR (HR: 5.56 [95%CI = 4.47–6.92]) were more likely to die within 2 years. Finally, compared to patients with chronic HCV infection, patients with alcoholic liver disease (HR: 1.19[95% CI = 1.11–1.28]) and decompensated cirrhosis (HR: 1.84 [95%CI = 1.73–1.96]) had a higher risk of death within 2 years after diagnosis of HCC.

**Table 3:**
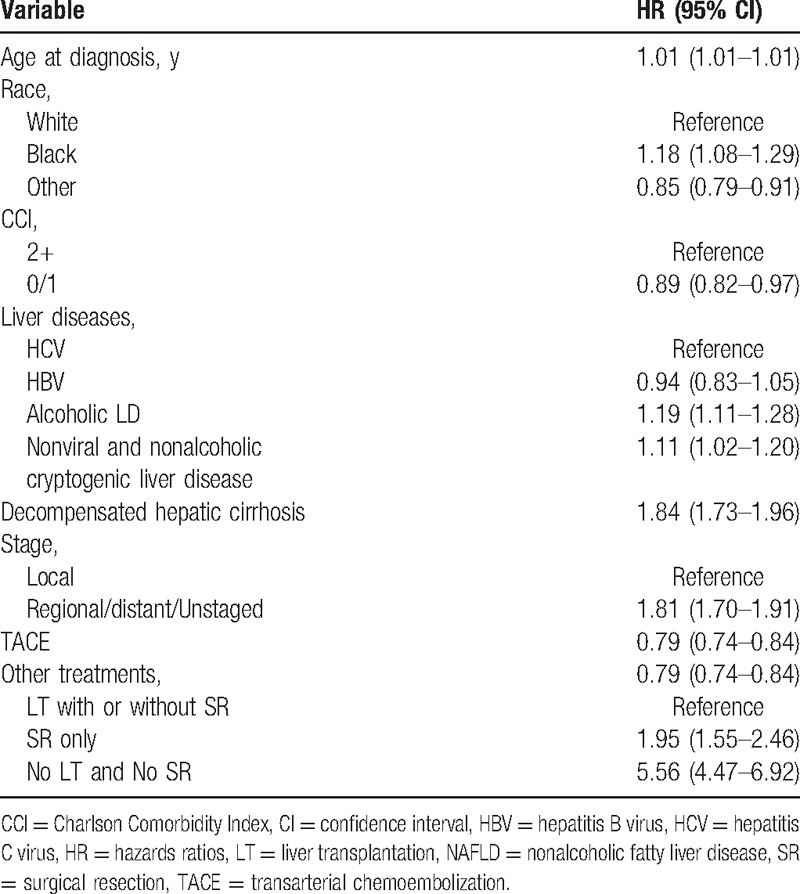
Multivariate adjusted hazards ratios (HRs) with 95% confidence intervals (CIs) for within 2 years.

### 3.4 Subgroup analysis

Since patients who underwent surgical resection outside HCC guidelines may affect their outcomes, we performed subgroup analysis to exclude these patients. In this subgroup analysis (n = 3523), we excluded (n = 7664) all those individuals with HCC who underwent surgical resection in the setting of decompensated cirrhosis or the presence of extrahepatic disease. In fact, after exclusion of these cases, the mortality within 2 years of diagnosis was similar between patients who received liver transplantation (25%) and surgical resection (34%, *P* value proportion = 0.85) as well as survival (*P* value log-rank = 0.25, Fig. 1).

## 4 Discussion

In the last few decades, hepatocellular carcinoma-related mortality has increased faster than mortality related to any other cancer types. Liver transplantation and surgical resection are the 2 potentially curative treatment options for patients with HCC,^[23–25]^ although deciding the right option may present a dilemma in some circumstances. Previous studies have revealed that disease-free survival, cancer recurrence rates, and mortality rates varied according to the selected treatment modality.^[16,18,20,21,26,27]^ The advantage of liver transplantation is that it not only can treat the HCC, but also the underlying cirrhosis. However, worldwide the shortage of donor organs has greatly limited the availability of LT.^[28–30]^ Compared to liver transplantation, surgical resection removes only HCC without dealing with the underlying liver disease, which limits its utility for HCC patients with advanced cirrhosis.

An interesting finding of our study was the proportions of types of liver disease and their associations with mortality among HCC patients. In our study, among the group with HCC, the prevalence of HCV, HBV, alcoholic liver disease, and nonviral nonalcoholic/cryptogenic liver disease was 52%, 9%, 21%, and 19%, respectively. In the United States, most patients with nonviral nonalcoholic/cryptogenic liver disease are represented by nonalcoholic fatty liver disease (NAFLD). These data suggesting HCV as the most common cause of HCC followed by alcoholic liver disease and NAFLD is consistent with previous reports.^[3,31–38]^ Interestingly, compared to patients with HCV-related HCC, HCC patients with alcoholic liver disease (1.19 times) and NAFLD (1.11 times) were more likely to die within 2 years of diagnosis, whereas HCC patients with HBV were less likely (0.94 times) to die 2 years after diagnosis. This finding is important, because, suggesting that NAFLD not only is among the top 3 causes of HCC but also is associated with higher mortality than HCV. In fact, these data are consistent with recent publication that suggested NAFLD-related HCC was associated with shorter survival time, more advanced tumor stage, and lower possibility of receiving liver transplantation than viral hepatitis-related HCC.^[38]^ Furthermore, although the prevalence of other liver diseases has remained stable, the prevalence of NAFLD is increasing.^[39]^ This increasing prevalence of NAFLD and its complications coupled with higher mortality of HCC related to NAFLD can cause significant future challenges.

As expected, our data showed that mortality within 2 years after diagnosis was significantly lower in the transplanted patients with HCC than those who received surgical resection only (30% vs 44%). In fact, compared to HCC patients who underwent surgical resection, although transplantation patients had significantly more severe disease (as reflected by higher Charlson scores and the presence of decompensated cirrhosis), these patients enjoyed lower mortality. This finding is consistent with previous literature describing superiority of liver transplantation over surgical resection.^[40,41]^ In a study of Dai et al, the outcomes of transplantation and surgical resection among patients with solitary HCC less than 8 cm were assessed. Despite a more compromised liver functions before the operation, no postoperative deaths occurred in the transplantation group, whereas there were 4 deaths in the surgical resection group. Within 1 year, mortality rates were 2% for the transplantation group and 5% for the surgical resection group. Finally, the rate of mortality in 3 years was 6% in transplanted patients, whereas it was 15% in surgical resection arm, suggesting a higher mortality associated with surgical resection.^[40]^ In our study, the multivariate analysis revealed that compared to patients undergoing liver transplantation, the risk of mortality within the first 2 years of diagnosis doubled for patients treated with surgical resection and increased more than 10 times in the absence of transplantation or surgery.

Another interesting finding of our study was that about 23% of patients with HCC who were treated with surgical resection had decompensated liver disease. Furthermore, 74% of patients with surgical resection had regional/distant/unstaged disease. Both of these findings suggest that a significant proportion of HCC patients who underwent surgical resection did not have early stage HCC or early stage cirrhosis which are recommended for resection per HCC treatment guidelines.^[18]^ These findings certainly could have impacted their survival and outcomes. It was previously reported that retrospective analysis of large-size surgical series showed that 43% to 48% of patients treated with surgical resection had diseases classified as intermediate or advanced stages according to the Barcelona Clinic Liver Cancer Group classification.^[42]^ For example, in a study of Ng et al,^[43]^ survival of patients who underwent surgical resection for early or intermediate HCC was compared. 48% of the study population was comprised of patients with intermediate-stage HCC (single nodule>5 cm or multinodular), who had significantly worse overall and disease-free survival rates and higher intrahepatic tumor recurrence rates than patients with early HCC. On the other hand, there is no doubt that patients with early, small HCC have excellent prognosis after hepatic resection when selected appropriately. Indeed, some previous studies have suggested that transplantation provides no significant advantage for patients with early staged HCC, whereas other studies have shown that survival after surgical resection was not inferior to transplantation in the appropriate patients.^[26,28,44–47]^ In a recent study, the outcomes of patients with early HCC (less than 2 cm, with Child Pugh A) were assessed. In this study, death within 2 years of diagnosis was 0% for both groups, whereas mortality within 3-year of diagnosis was 8.3% for transplantation and 6.7% for resection.^[28]^ In our analysis, we assessed the impact of “appropriate surgical resection” by performing a subgroup analysis that focused on the outcome of surgical resection within “guideline” recommendation. In fact, mortality rates for the group that underwent surgical resection and liver transplantation were 34% and 25% (*P* = 0.85). This analysis showed that the mortality rate of surgical resection decreases substantially when patients receive surgical resection appropriately in the absence of decompensated cirrhosis and distant staged disease. In fact, these findings are consistent with previous studies highlighting the importance of appropriate selection of patients for surgical resection.^[20,44,48,49]^

There are a number of limitations to this study. First, even though we provided data from a strong population-based database, the linkage between SEER data and Medicare can be limited. Usually all cases reported by the SEER registries are matched to the Medicare database, but, depending on the year of a diagnosis, the linked data may not show up. Second, although Medicare is a large database and frequently used by healthcare researchers, ICD-9 codes have been used for disease definitions and there might be discrepancies in the accuracy and the completeness of the data.^[50]^ Also, as there are multiple ICD-9 codes for a particular disease, and patients may have more than 1 underlying liver conditions, there is a possibility of minor ineluctable overlaps in disease categories. Finally, we had 24-months of follow-up and survival data for the study population, which might be insufficient to assess the differences in survival rates among these groups.

In conclusion, within 2 years mortality was significantly lower in the transplantation group than the surgical resection group. However, once “inappropriate” cases of surgical resections were removed, the survival was similar in both groups. We also conclude that compared to patients with HCV-related HCC, patients with alcoholic liver disease-related HCC and NAFLD related HCC are more likely to die within 2 years. As the prevalence of obesity and related NAFLD increases, this will be an important issue in the context disease burden related to HCC in the United States.

## Acknowledgments

The authors would like to thank Deena Hallaji, Manirath Srishord, and Brian Lam, PA-C for their great support during the formation of the manuscript.

## Supplementary Material

Supplemental Digital Content
