# Supplementary material for: Mortality assessment of patients with hepatocellular carcinoma according to underlying disease and treatment modalities
Source: Medicine (Baltimore). 2017 Mar 3;96(9):e5904. doi: 10.1097/MD.0000000000005904 (PMC5340426; doi:10.1097/MD.0000000000005904)
Supplement: Supplemental Digital Content [file medi-96-e5904-s001.docx]

Mortality Assessment of Patients with Hepatocellular Carcinoma According to Underlying Disease and Treatment Modalities

Pegah Golabi

Supplementary Digital Content: Characteristics of study by liver transplantation (LT) and surgical resection (SR) status in pair-wise matched ^1^ cohorts, SEER-Medicare, 2001-2009

| **Variable** | **LT vs SR only** | | **LT vs Control** | | **SR only vs Control** | |
| --- | --- | --- | --- | --- | --- | --- |
|  | **LT**  **(n = 298)** | **SR only**  **(n = 552)** | **LT**  **(n = 296)** | **Control**  **(n = 556)** | **SR only**  **(n=2150)** | **Control**  **(n=3645)** |
| **Age at diagnosis (years): mean (SD)** | 61.33 (8.34) | 62.01 (8.57) | 61.40 (8.34) | 61.09 (9.89)* | 71.27 (8.66) | 71.42 (9.81) |
| **Died within two years** | 87 (29.2%) | 204 (37.0%)* | 86 (29.1%) | 408 (73.4%)* | 948 (44.1%) | 2948 (80.9%) |
| **Male** | 216 (72.5%) | 402 (72.8%) | 213 (72.0%) | 440 (79.1%)* | 1434 (66.7%) | 2506 (68.8%) |
| **Race,** |  |  |  |  |  |  |
| White | 225 (75.5%) | 347 (62.9%)* | 223 (75.3%) | 366 (65.8%)* | 1386 (64.5%) | 2404 (66.0%)* |
| Black | 25 (8.4%) | 82 (14.9%)* | 24 (8.1%) | 85 (15.3%)* | 192 (8.9%) | 424 (11.6%)* |
| Other | 48 (16.1%) | 123 (22.3%)* | 49 (16.6%) | 105 (18.9%)* | 572 (26.6%) | 817 (22.4%)* |
| **CCI,** |  |  |  |  |  |  |
| 0/1 | 280 (94.0%) | 354 (64.1%)* | 278 (93.9%) | 386 (69.4%)* | 1371 (63.8%) | 2558 (70.2%)* |
| 2+ | 18 (6.0%) | 198 (35.9%)* | 18 (6.1%) | 170 (30.6%)* | 779 (36.2%) | 1087 (29.8%)* |
| **Liver diseases,** |  |  |  |  |  |  |
| HCV | 213 (73.4%) | 300 (66.8%)* | 210 (72.9%) | 257 (63.3%)* | 810 (55.9%) | 1185 (52.0%)* |
| HBV | 12 (4.1%) | 34 (7.6%) | 12 (4.2%) | 26 (6.4%) | 170 (11.7%) | 184 (8.1%)* |
| Alcoholic LD | 34 (11.7%) | 57 (12.7%) | 35 (12.2%) | 71 (17.5%)* | 215 (14.8%) | 477 (20.9%)* |
| Non-viral and non-alcoholic cryptogenic liver disease (NAFLD) | 31 (10.7%) | 58 (12.9%) | 31 (10.8%) | 52 (12.8%)* | 255 (17.6%) | 433 (19.0%)* |
| **Decompensated hepatic cirrhosis** | 237 (79.5%) | 155 (28.1%)* | 236 (79.7%) | 214 (38.5%)* | 487 (22.7%) | 1290 (35.4%)* |
| **Stage,** |  |  |  |  |  |  |
| Local | 225 (75.5%) | 405 (73.4%) | 222 (75.0%) | 403 (72.5%) | 583 (27.1%) | 1133 (31.1%) |
| Regional/distant/Un-staged | 73 (24.5%) | 147 (26.6%) | 74 (25.0%) | 153 (27.5%) | 435 (20.2%) | 664 (18.2%) |
| **Treatments,** |  |  |  |  |  |  |
| TACE | 108 (36.2%) | 197 (35.7%) | 107 (36.1%) | 175 (31.5%) | 618 (28.7%) | 1103 (30.3%) |

^1^ Propensity score (1:2 frequency) matched based on age (years), tumor stage, and HCC diagnosis year;

* Significant (P < 0.05) for pair-wise comparisons between matched cohorts
